# Enemas with sucralfate and n-acetylcysteine can reduce inflammation and oxidative stress in colonic mucosa without fecal stream

**DOI:** 10.1590/acb406325

**Published:** 2025-08-18

**Authors:** Marcelo César Zanesco, Mateus Magami Yoshitani, Felipe Leonardo Fagundes, Fábio Guilherme Campos, Poliana Pacciulli Pereira, Quélita Cristina Pereira, José Aires Pereira, Raquel de Cássia Santos, Carlos Augusto Real Martinez

**Affiliations:** 1 Universidade São Francisco – Postgraduate Program in Health Sciences – Bragança Paulista (SP) – Brazil.; 2 Universidade São Francisco – Faculty of Medicine – Bragança Paulista (SP) – Brazil.; 3 Universidade Estadual Paulista – Postgraduate Program in General and Applied Biology – Botucatu (SP) – Brazil.; 4 Universidade de São Paulo – Faculty of Medicine – Department of Gastroenterology – São Paulo (SP) – Brazil.; 5 Universidade Estadual de Campinas – Department of Surgery – Postgraduate Program in Surgical Sciences – Campinas (SP) – Brazil.

**Keywords:** Fatty Acids, Volatile, Colitis, Oxidative Stress, Sucralfate, Acetylcysteine, Models, Animal

## Abstract

**Purpose:**

To evaluate whether enemas containing sucralfate (SCF) alone or in combination with n-acetylcysteine (NAC) reduces inflammation and oxidative stress (OS) in the colonic mucosa without fecal stream.

**Methods:**

Forty-eight rats were subjected to left colostomy and distal rectal mucous fistula. During the procedure, 2 cm of the colon was collected to constitute the sham group. Twelve weeks after the surgical procedure, the animals were divided into two groups (n = 24) and received daily enemas containing saline, SCF (2 g/kg), NAC (100 mg/kg), or SCF + NAC (2 g/kg + 100 mg/kg, respectively) for two or four weeks. At the end of the intervention period, the animals were euthanized, and colonic segments without fecal stream were removed for histological and biochemical analyses. The diagnosis of colitis was made by histological analysis, and the inflammatory score was assessed using a validated scale. The neutrophilic infiltrate was evaluated by quantifying the content of myeloperoxidase (MPO) in the tissue. OS was determined by evaluating the activity of colonic antioxidant systems (superoxide dismutase, catalase, and reduced glutathione) and malondialdehyde (MDA) levels. The differences among subgroups were analyzed with the Mann-Whitney’s test, whereas changes over time were analyzed via the Kruskal-Wallis’ test, with the significance level of 5% (*p* < 0.05).

**Results:**

Enemas with SCF and NAC alone or in combination reduced colonic inflammation and the tissue levels of MPO and MDA and increased the levels of antioxidant enzymes.

**Conclusion:**

SCF and NAC enemas alone or in combination reduced inflammation activity and OS in colon segments without fecal stream.

## Introduction

Glotzer et al.^1^ first described the development of an inflammatory process in the mucosa of colon segments without fecal stream. They named this new form of colorectal inflammatory disease diversion-related colitis^1^. Since this initial description, studies have demonstrated that the etiopathogenesis of diversion colitis (DC) is likely related to intraluminal deficiency of short-chain fatty acids (SCFAs), which are the main energy substrate for colon epithelial cells^2^. When the cells of the colonic epithelium stop receiving their main energy fuel, they undergo important changes in the mechanisms for energy production (respiratory metabolic chain), and they begin to produce a large quantity of reactive oxygen species (ROS)^3^. These ROS likely cause damage to colonic mucosa cells, leading to the development of oxidative stress (OS)-induced damage to the epithelium and triggering the development of the inflammatory process of the colonic mucosa that characterizes DC^3^.

The role of ROS in the development of DC was confirmed by the results of several experimental studies showing that OS represents one of the main molecular mechanisms related to the first steps in the etiopathogenesis of DC^4^. ROS can damage several mechanisms of defense that function at the epithelial barrier, such as the mucous layer that covers the colonic mucosa, proteins that participate in cellular adhesion molecular systems, and can destroy the basal cell membrane^5^–^8^. Corroborating this possibility, the application of substances with antioxidant activity in experimental models of DC has been shown to reduce colonic mucosa inflammation and the ROS-induced damage to the different mechanisms of defense that function at the colonic epithelial barrier^6^–^14^.

Sucralfate (SCF) and n-acetylcysteine (NAC) are two substances with important antioxidant activity and have been used with good results in different forms of colitis, such as radiation proctitis, ulcerative colitis, and DC^6^,^8^–^14^. Experimental studies have demonstrated that the application of daily enemas containing SCF or NAC can reduce colonic mucosa inflammation, as well as the tissue levels of ROS in models of DC^6^,^14^. However, to the best of our knowledge, no study has evaluated whether the application of enemas containing SCF and NAC alone or in combination is more effective at reducing OS in an experimental model of DC.

Thus, the aim of the present study was to evaluate the effectiveness of applying enemas with both substances alone or in combination in reducing inflammation, the neutrophil infiltrate, and OS in an experimental model of DC.

## Methods

### Ethics

The experiments were performed in accordance with the principles outlined by Federal Law no. 11.794 (10/08/2008) and approved by the Ethics Committee in Animal Research of Universidade São Francisco (no. 006.06.2020).

Forty-eight male specific pathogen-free Wistar rats (300–350 g) were obtained from the Faculty of Medicine of the Universidade São Francisco barrier facility and maintained on light/dark cycles of 12 hours; they were fed a standard rodent diet. The rats were deprived of food, but not water, for 12 h prior to the surgical procedure.

### Surgical techniques

The diversion of the fecal stream was performed according to the previous validated experimental model of DC^3^–^5^. All the animals underwent surgery with general anesthesia via the intramuscular administration of 0.1 mL/100 g of a 1:1 (v/v) solution of ketamine (50 mg/mL) and xylazine (20 mg/mL). The abdomen was shaved, and a 4-cm-long midline incision was made. In all the animals, a 2-cm-long segment of the left colon located 9 cm above the Peyer’s lymphoid patch was collected to constitute the sham group (colon with fecal stream). The remaining segment of the left colon was then exteriorized through a circular skin hole 3 mm in diameter (proximal colostomy). The distal segment of the colon was exteriorized through another caudal skin orifice. The proximal and distal stomas were fixed to the skin with a separate stitch of nylon (Monolylon-Ethilon-5-0, Ethicon, Brazil). Before the distal stoma was fixed to the skin, the distal colon was carefully cleaned with an infusion of 37°C saline until the residual fecal contents were completely removed. The abdominal incision was closed in two stages (aponeurosis and skin) with uninterrupted sutures of nylon (Mononylom-Ethilon 3-0, Ethicon, Brazil). In this way, two colostomies were performed: one proximally as a terminal colostomy with fecal stream and a second stoma as a distal mucosa fistula (segment without fecal stream). Analgesic (acetaminophen) was administered via the drinking water for three days after the surgical procedure. The rats were maintained in individual cages for 12 weeks until they developed DC, and no particular care was taken with respect to the stomas and abdominal incisions. Two animals died postoperatively after intestinal transit diversion and were replaced.

### Experimental groups

**Figure 1:**
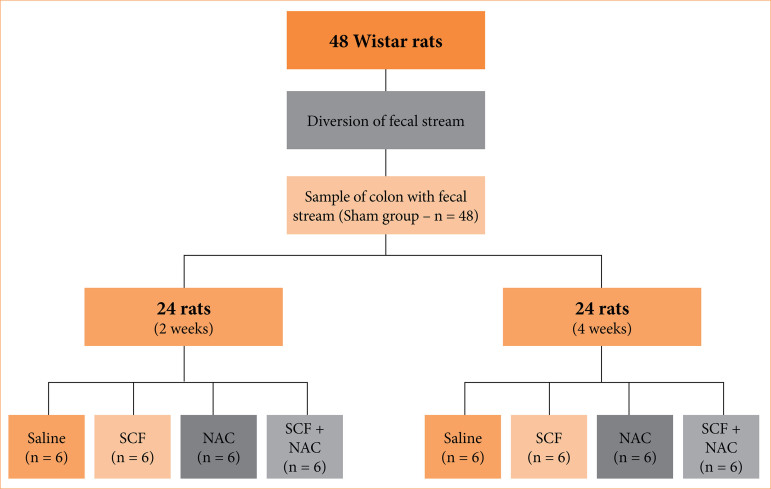
Division of the experimental groups.

SCF: sucralfate; NAC: n-acetylcysteine. Source: Elaborated by the authors.

Once the surgical procedure was completed, all the animals were maintained in individual cages for 12 weeks to ensure the development of DC. The 48 animals were divided into two main experimental groups with 24 animals each, according to which they were euthanized after two or four weeks. Each experimental group of 24 rats was divided into four subgroups (n = 6) according to the intervention solution administered. Six animals in each group received daily rectal enemas with 10 mL/day of saline (control subgroup) at 37°C for two (n = 6) or four weeks (n = 6). In the second subgroup, six animals received daily rectal enemas with 10 mL of SCF (Sigma-Aldrich, St. Louis, MO, United States of America) at the concentration of 2 g/kg/day for two (n = 6) or four weeks (n = 6). Animals in the third subgroup received daily enemas with 10 mL of NAC (Sigma-Aldrich Brasil LTDA, São Paulo, SP, Brazil) at the concentration of 100 mg/kg/day for two (n = 6) or four weeks (n = 6). Finally, the animals in the fourth group received daily enemas containing an emulsion supplemented with 10 mL of SCF (2 g/kg/day) and NAC (100 mg/kg/day). Figure 1 summarizes the experimental design of this study.

In both experimental groups that received NAC, NAC was dissolved in phosphate buffered saline (PBS) solution, and its acidic pH was adjusted with NaOH (2 M) to pH 7.4. To standardize the rate and time of application, enemas were administered to all animals with an infusion pump, with the pump rate standardized to 2 mL/min.

### Sample collection

Upon the completion of the predetermined irrigation period, the animals were anesthetized as described above, and the midline incision was opened again. In all groups, after the conclusion of the intervention period (two or four weeks), the entire segment of the colon without fecal stream subjected to irrigation with the proposed substances was removed. The removed colon segment, measuring approximately 4 cm, was longitudinally opened through the antimesenteric border and divided into two longitudinal segments. The first segment was fixed on a piece of cork and subjected to histological and histochemical analysis. The second removed segment of the colon was divided into five parts, placed in five Eppendorf tubes and immediately stored in a freezer (-80°C) for subsequent biochemical assays to measure the tissue levels of myeloperoxidase (MPO) and malondialdehyde (MDA), and the activity of the antioxidant systems catalase (CAT), superoxide dismutase (SOD), and reduced glutathione (GSH). This standardization of material collection for histological and biochemical assays was used in all animals.

### Histological analysis

Fragments prepared for histological analysis were immersed in 10% neutral formalin buffer (Sigma-Aldrich, St. Louis, MO, United States of America) for 24 h, dehydrated by exposure to increasing ethanol concentrations, and embedded in paraffin. Thereafter, sections of tissue were cut at 5-µm thickness on a rotary microtome (Leica Biosystems, Nussloch, Germany), mounted on a glass slide, cleared, hydrated and stained with hematoxylin‒eosin (HE) to evaluate the presence of colitis. Slide analysis was performed using an optical microscope (Eclipse DS-50, Nikon Inc., Osaka, Japan) with a final magnification of 200×. Photomicrographs were collected with a digital video-capture camera (DS-Fi-50; Nikon Inc., Osaka, Japan) coupled to the microscope body and digitized by a computer system for image analysis (NIS-Elements; Nikon Inc., Osaka, Japan). The diagnosis of colitis was made by two different pathologists according to the presence of three independent histological parameters: reduction in crypt length, neutrophil infiltration into the mucosa, and epithelial loss. These variables were stratified according to the degree of each as follows:

- + absent or no alterations;
- ++ mild alterations;
- +++ moderate alterations;
- ++++ intense alterations.

For all the analyzed variables, the final value considered for each animal was the mean value after quantification of three distinct histological fields.

### Myeloperoxidase activity

To evaluate the MPO activity in the frozen colonic tissue samples, a small fragment of the tissue was homogenized in 0.5% (w/v) hexadecyltrimethylammonium bromide in 50 mM potassium phosphate buffer, pH 6. For the MPO assay, 50 mL of each sample was added to 200 mL of o-dianisidine solution (0.167 mg/mL o-dianisidine dihydrochloride and 0.0005% hydrogen peroxide in 50 mM phosphate buffer, pH 6). Immediately afterwards, the change in absorbance was read at 460 nm over a 5-min period using a microplate reader (Multiscan MS; Labsystems, Joensuu, Finland). MPO activity was assessed as an index of neutrophil infiltration^15^.

### Determination of malondialdehyde levels

The levels of lipid peroxidation were evaluated by measuring the levels of thiobarbituric acid reactive substances (TBARS), as with MDA, with a previously described methodology. MDA is a secondary product of lipid oxidation and is considered a candidate general biomarker of OS. To quantify the tissue levels of MDA, 1 g of each frozen fragment was placed in 5 mL of phosphate buffer and homogenized by vertexing and ultrasound sonication for 30 s; this process was repeated three times. Then, 250 µL of the supernatant obtained from the homogenization process was transferred to a plastic test tube containing 25 mL of 4% methanolic BHT, followed by another round of homogenization by vortexing. The sample was then mixed with 1 mL of 12% trichloroacetic acid, 1 mL of 0.73% thiobarbituric acid, and 750 µL of Tris/HCl buffer and then incubated in a water bath at 100°C for 60 min. After this step, the tubes were immediately placed in a container with ice to block the reaction, with the addition of 1.5 mL of n-butanol. The mixture was then vortexed again for 30 s. The samples were separated by centrifugation for 10 min at 5,000 rpm. Finally, the supernatant was removed, and the absorbance of the organic phase at 532 nm was analyzed with a ultraviolet/vis 6105 (Jenway, Bibby Scientific Limited, Cheshire, United Kingdom) spectrophotometer.

### Catalase activity

Colon tissue samples (50 mg) were homogenized in extraction buffer–1% Triton X-100, 100 mM Tris-HCl (pH 7.4), 100 mM sodium pyrophosphate, 100 mM sodium fluoride, 10 mM EDTA, 10 mM sodium orthovanadate, 2.0 mM PMSF, and 0.1 µg/mL aprotinin–at 4°C, followed by centrifugation at 14,000 rpm for 45 minutes. The supernatant was diluted 1:20 with PBS, and 5 µL of this mixture was added to a 96-well plate with 100 µL of assay buffer (250 mM KH₂PO₄, pH 7), 30 µL of methanol, and 20 µL of hydrogen peroxide (35.3 mM). The plate was incubated at room temperature for 20 minutes with agitation. Subsequently, 30 µL of hydrogen hydroxide (10 M) and 45 µL of Purpald reagent (34.2 mM) were added, followed by 10 minutes of incubation. Then, 15 µL of potassium periodate (65.2 mM) was added, and the plate was incubated for an additional 5 minutes in the dark. The absorbance was measured at 540 nm, and CAT activity was quantified using a formaldehyde standard curve (0–75 µM) and normalized to the total protein content in the undiluted samples.

### Superoxide dismutase activity

The frozen tissue samples were thawed, subsequently homogenized as described previously, and diluted 1:10 (15 µL sample + 285 µL PBS), followed by centrifugation at 12,000 rpm for 15 minutes at 4°C. The supernatant was placed into a 96-well plate, and 150 µL of a solution containing hypoxanthine, xanthine oxidase, and nitroblue tetrazolium chloride (1:1:1, v/v) was added to each well. The absorbance was measured at 560 nm for 10 minutes, with readings taken at 1-minute intervals. SOD activity was calculated by determining the area under the curve (AUC) of the absorbance data, which was normalized to the total protein content and adjusted by the dilution factor.

### Reduced glutathione levels

Stored colon samples were homogenized in extraction buffer and diluted 1:20 (30 µL sample + 270 µL PBS), followed by centrifugation at 12,000 rpm for 15 minutes at 4°C. One hundred microliters of the supernatant were added to a 96-well plate containing Tris-EDTA buffer (1 mM/2 mM, pH 8.2). Then, 100 µL of a standard curve solution containing L-reduced glutathione (0–500 nmol/mL) and 100 µL of sample were added, and an initial absorbance reading was taken at 412 nm. Afterwards, 20 µL of 5,5’-dithiobis (2-nitrobenzoic acid) (DTNB, 10 mM in methanol) was added, and the plate was incubated at room temperature for 15 minutes in the dark. A second absorbance reading was taken at the same wavelength. GSH levels were determined by subtracting the initial absorbance from the post-DTNB absorbance and using a standard curve. The resulting values were multiplied by the dilution factor, normalized to the total protein content, and expressed as nmol GSH/mg protein. The protein concentration was determined by the biuret method using the Protal Kit (Laborclin, Minas Gerais, Brazil).

### Statistical analysis

The degree of inflammation was described according to the median of the values obtained in each experimental group. The tissue levels of MPO, MDA, CAT, SOD, and GSH are presented as the means ± standard errors. Comparisons of the results between the saline and SCF, NAC, and SCF + NAC groups during the same period of intervention were performed via the Mann‒Whitney’s t test. The Kruskal‒Wallis’ test was used to analyze the changes in the results of treatment with each substance over intervention time. For all tests, the level of significance of 5% was established (*p* < 0.05). One asterisk (*) was used to identify values of *p* < 0.05, and two asterisks (**) were used for values of *p* < 0.01 when the results in the intervention group were compared with those in the saline group within the same intervention period (two or four weeks). One cross (†) was used to identify values of *p* < 0.05, and two crosses (††) were used for values of *p* < 0.01 when we compared the intervention with one of the substances used (saline, SCF, NAC, or SCF + NAC) after two or four weeks of intervention.

## Results

**Figure 2:**
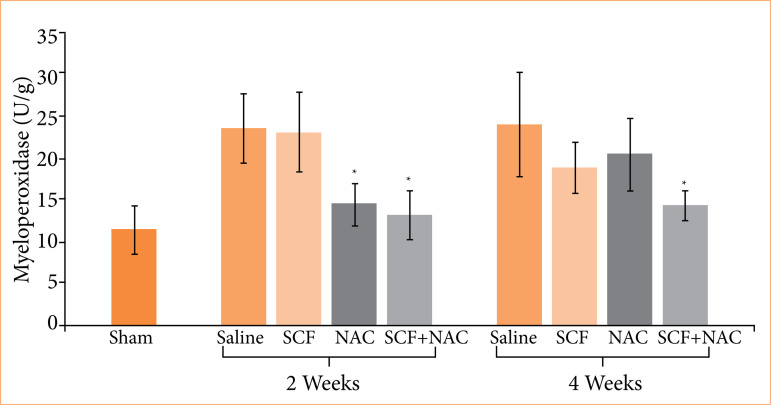
Comparison of serum levels of myeloperoxidase (MPO) in units per gram (U/g) in the sham subgroup and in animals treated with saline, SCF, NAC or SCF + NAC for two or four weeks.

SCF: sucralfate; NAC: n-acetylcysteine; *p < 0.05 according to the Mann‒Whitney’s test. Source: Elaborated by the authors.

Figure 2 shows the mean values, with the respective standard errors, of the serum levels of MPO in units per gram of tissue (U/g), and the values of the different experimental subgroups at two treatment time points were compared (two or four weeks).

**Figure 3:**
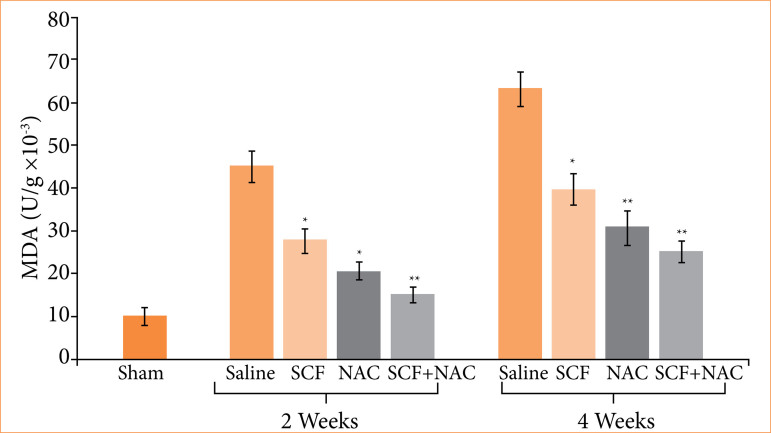
Comparison of serum levels of malondialdehyde (MDA) in units per gram (U/g × 10^-3^) in the sham subgroup and in animals treated with saline, SCF, NAC or SCF + NAC for two or four weeks.

SCF: sucralfate; NAC: n-acetylcysteine; *p < 0.05 according to the Mann‒Whitney’s test; **p < 0.01 according to the Mann‒Whitney’s test. Source: Elaborated by the authors.

Figure 3 shows the mean values, with their respective standard errors, of the tissue levels of MDA, in units per gram of tissue (U/g × 10^-3^), and the values of the different experimental subgroups at two treatment time points were compared (two or four weeks).

**Figure 4:**
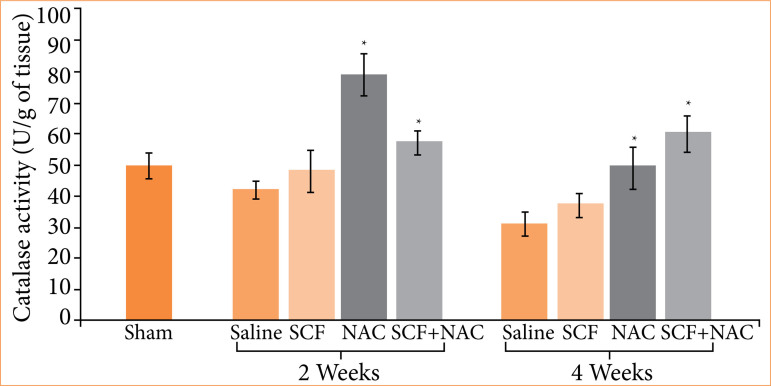
Comparison of tissue catalase levels in units per gram (U/g) in the sham subgroup and in animals treated with saline, SCF, NAC or SCF + NAC for two or four weeks.

SCF: sucralfate; NAC: n-acetylcysteine; *p < 0.05 according to the Mann‒Whitney’s test. Source: Elaborated by the authors.

Figure 4 shows the mean values, with their respective standard errors, of tissue CAT levels, in units per gram of tissue (U/g), and the values of the different experimental subgroups at two treatment time points were compared (two or four weeks).

**Figure 5:**
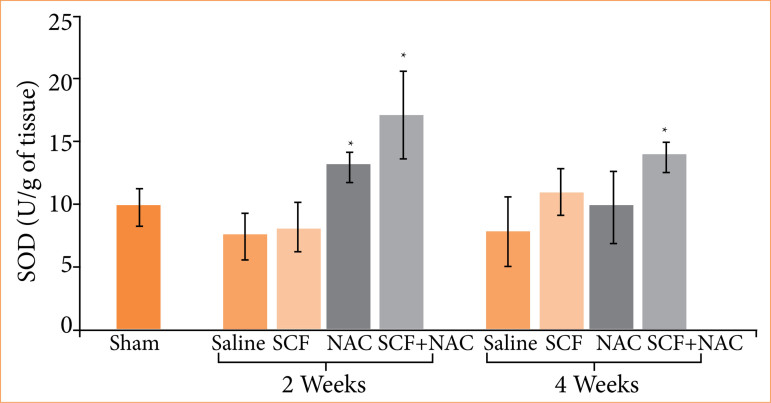
Comparison of tissue superoxide dismutase (SOD) levels in units per gram (U/g) in the sham subgroup and in animals treated with saline, SCF, NAC or SCF+NAC for two or four weeks.

SCF: sucralfate; NAC: n-acetylcysteine; *p < 0.05 according to the Mann‒Whitney’s test. Source: Elaborated by the authors.

Figure 5 shows the mean values, with respective standard errors, of tissue SOD levels, in units per gram of tissue (U/g), and the values of the different experimental subgroups at two treatment time points were compared (two or four weeks).

**Figure 6:**
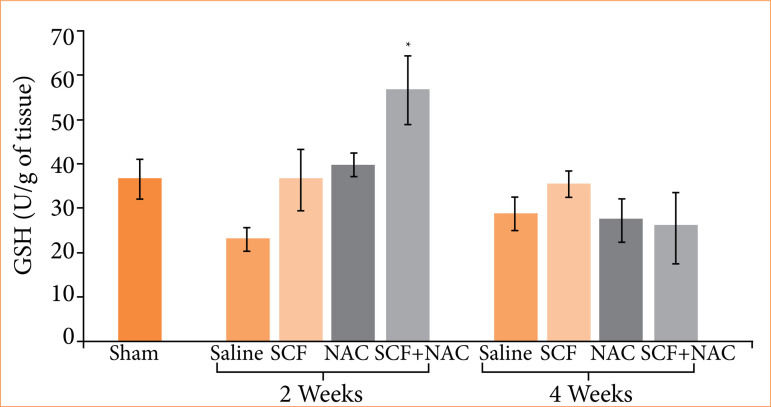
Comparison of tissue glutathione (GSH) levels in units per gram (U/g) in the sham subgroup and in animals treated with saline, SCF, NAC and SCF + NAC for two or four weeks.

SCF: sucralfate; NAC: n-acetylcysteine; *p < 0.05 according to the Mann‒Whitney’s test. Source: Elaborated by the authors.

Figure 6 shows the average values, with their respective standard errors, of the tissue levels of GSH, in units per gram of tissue (U/g), and the values of the different experimental subgroups at two treatment time points were compared (two or four weeks).

**Figure 7:**
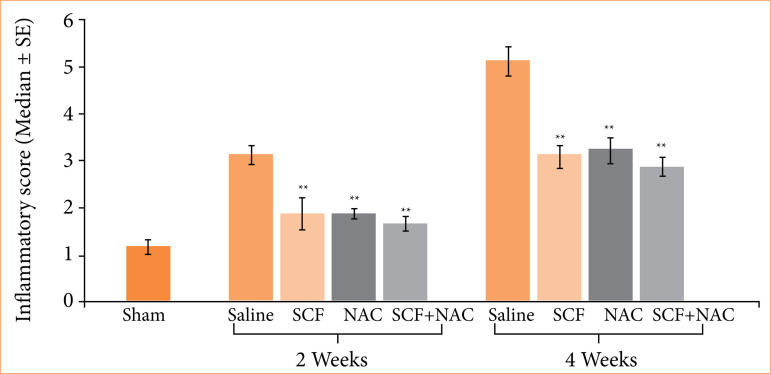
Comparison of inflammatory scores (medians ± SEs) in the sham subgroup and in animals treated with saline, SCF, NAC or SCF + NAC after two or four weeks of intervention.

SCF: sucralfate; NAC: n-acetylcysteine; SE: standard error; **p < 0.01 according to the Mann‒Whitney’s test. Source: Elaborated by the authors.

Figure 7 shows the mean values, with their respective standard errors, of the inflammatory score, and the values of the different experimental subgroups at two treatment time points were compared (two or four weeks).

Table 1 shows the differences in the tissue activities of MPO (U/g), MDA (U/g ×10-3), CAT (U/g), SOD (U/g), and GSH (U/g) and the median inflammatory score (Md), with the respective standard errors, between groups subjected to interventions for two or four weeks.

**Table 1:** Variation in mean levels of myeloperoxidase (MPO) (U/g), malondialdehyde (MDA) (U/g × 10^-3^), catalase (CAT) (U/g), superoxide dismutase (SOD) (U/g), glutathione (GSH) (U/g), and inflammatory score (median), with the respective standard errors, in groups subjected to interventions for two or four weeks.

	Two weeks						Four weeks			
	Sham	Saline	SCF	NAC	SCF + NAC		Saline	SCF	NAC	SCF + NAC
I.S. (M ± SE)	1.1 ± 0.1	3.12 ± 0.2	1.87 ± 0.3	1.88 ± 0.1	1.66 ± 0.1		5.11 ± 0.2†	3.11 ± 0.2†	3.22 ± 0.2†	2.88 ± 0.1†
MPO (U/g)	145 ± 23	220 ± 9	188 ± 9	198 ± 25	179 ± 21		245 ± 18	160 ± 2	115 ± 15†	85 ± 6.1††
MDA (U/g × 10^-3^)	10.12 ± 2.1	45.1 ± 3.7	27.7 ± 3.9	26.6 ± 2.3	15.2 ± 1.2		63.2 ± 4.2††	39.5 ± 3.2†	30.7 ± 4.2†	25.1 ± 2.6†
CAT (U/g)	50 ± 4.2	42 ± 3.2	48 ± 7.1	79 ± 7.2†	57 ± 4.2		31 ± 4.1	37 ± 4.0	49 ± 7.3	60 ± 6.1
SOD (U/g)	9.9 ± 1.5	7.5 ± 1.9	8.2 ± 2.9	13.1 ± 1.2†	17.2 ± 3.6†		7.9 ± 2.8	11 ± 1.9	9.8 ± 2.9	13.9 ± 1.3
GSH (U/g)	36.5 ± 4.5	23.0 ± 2.9	36.4 ± 7.0	39.9 ± 2.8†	56.7 ± 7.9†		28.8 ± 3.9	35.6 ± 3.1	27.3 ± 5.1	25.8 ± 8.1

I.S.: inflammatory score; M: mean; SE: standard error; SCF: sucralfate; NAC: n-acetylcysteine; MPO: myeloperoxidase; MDA: malondialdehyde; CAT: catalase; SOD: superoxide dismutase; GSH: reduced glutathione; U/g: units per gram; Kruskal‒Wallis’ test with Dunn’s posttest; †*p* < 0.05; ††*p* < 0.01. Source: Elaborated by the authors.

## Discussion

DC is an inflammatory disease that develops in segments of the colon and rectum that are devoid of fecal stream^1^. Clinical and experimental studies suggest that the etiopathogenesis of DC is related to the lack of a continuous supply of SCFAs due to intestinal transit diversion^3^,^4^. SCFAs (acetate, propionate, and butyrate) are formed from the fermentation of insoluble fibers ingested in the diet. Specialized bacteria that are present mainly in the lumen of the colon metabolize these dietetic fibers to form SCFAs. Butyrate is absorbed by cells of the colonic epithelium, mainly in the proximal colon, via passive diffusion. In the distal colonic segment of the colon, butyrate is absorbed by active transport mechanisms. More recently, studies have shown that the diversion of intestinal transit can cause marked changes in the microbiota that is present inside the colonic lumen. These changes reduce the fermentation of dietary fibers and, consequently, the production of SCFAs^16^,^17^.

The results of the present study seem to support this evidence. In segments with fecal stream that were collected during the surgical procedure performed for intestinal diversion (sham group), the degree of inflammation, as shown by the histological study and by the tissue levels of MPO, was always lower than that in the animals treated with saline alone, regardless of the irrigation time. The OS levels, as shown by the tissue content of MDA, were always lower in the colonic segments in which fecal transit was preserved. In other words, maintaining fecal transit reduced the formation of ROS and, consequently, the degree of tissue damage caused by OS. There were also higher tissue activities of the three antioxidant systems analyzed (CAT, SOD, and GSH) in the segments with a maintained fecal stream than in the animals that received saline alone. These findings support the importance of a regular supply of SCFAs to maintain the homeostasis of cellular energy metabolism and, consequently, prevent the overproduction of ROS^16^,^17^.

The overproduction of ROS is toxic to the colonic epithelium and can damage several mechanisms of defense employed by the healthy colonic epithelial barrier^3^–^7^. Thus, DC can be considered a disease resulting from an energy deficiency syndrome, and the initial damage to the colonic epithelial barrier can be related to the overproduction of ROS due to alterations in cellular energetic metabolism^18^.

Several strategies for the treatment of DC have been proposed, although none have been definitively established. Treatment approaches using enemas containing SCFAs, 5-aminosalicylic acid, steroids, somatostatin, fecal transplant, SCF, and NAC in diverted colonic segments show different degrees of efficacy in mitigating mucosal inflammation and oxidative tissue damage^6^,^8^,^13^,^14^,^18^–^20^.

Among the various strategies proposed for the treatment of DC, the application of enemas containing SCF and NAC has shown promising results, not only by reducing the mucosal inflammatory process and OS, but also by preventing the breakdown of the colonic epithelium’s defense mechanisms by ROS^6^,^8^,^13^,^14^. The results found in the present study corroborated this evidence when we verified that the application of enemas using SCF and NAC in isolation were capable of reducing the levels of MDA, a recognized marker of lipid peroxidation of cell membranes and which reflects the intensity of tissue oxidative stress. However, regarding antioxidant systems, only animals submitted to intervention with NAC increased tissue levels of CAT and SOD, mainly after two weeks, suggesting that NAC has greater antioxidant activity when compared to SCF. Glutathione peroxidase levels did not change in animals submitted to interventions with both substances, suggesting that this tissue antioxidant system is less impacted by drugs.

SCF has been also used to improve the symptoms of severe radiation proctitis^9^,^10^. The mechanism of action of SCFs is related to their high capacity for adhesion to inflamed and ulcered epithelium^21^. However, the cytoprotective action of SCF on the epithelium of the digestive tract is more complex and is related to different mechanisms of action^21^. Kochhar et al.^22^ were the first authors to demonstrate the effectiveness of applying enemas containing SCF in controlling rectal bleeding resulting from radiation proctitis. Posteriorly, the beneficial effects of enemas with SCF were used to treat other forms of inflammatory bowel diseases that led to the formation of epithelial ulcers^23^. Using experimental models of DC, it was shown that the application of enemas with SCF could reduce the inflammatory process in the colonic mucosa excluded from fecal stream, protecting against the different mechanisms of defense that function at the epithelial mucosal barrier, and decreasing the levels of tissue OS^8^,^13^,^14^,^24^.

The topical application of SCF also has antioxidant activity and can neutralize the formation of ROS produced by neutrophils present in inflamed tissue. This antioxidant action protects against the peroxidation of lipids that are components of cell membranes, the second line of epithelial defense, protecting the gastrointestinal mucosa against tissue OS^25^. When we evaluated the individual effects of the application of enemas containing SCF in this experimental study, we found that SCF reduced MDA levels regardless of the intervention time, indicating that SCF has antioxidant activity. These findings were confirmed when the tissue levels of antioxidant system components (CAT, SOD, and GSH) were shown to be maintained. Thus, the reduction in the levels of MDA in the colonic mucosa cells was related to the use of SCF. These findings may be related not only to its antioxidant activity but also to its high capacity for adhesion to and protection of the inflamed mucosa.

NAC is another substance with recognized antioxidant activity both *in vitro* and *in vivo* and has been used for the treatment of various inflammatory diseases^6^,^12^,^26^–^29^. NAC is considered a molecule with great antioxidant activity in the human body and has several therapeutic actions. Shirazi et al.^27^ confirmed that, compared with placebo, NAC has a significantly positive effect on maintaining remission of attacks in patients with ulcerative colitis who are in the reduction phase of steroid therapy.

Studies with chemically induced colitis models have shown that NAC improves the mucosal inflammatory process by removing ROS formed during cellular energy metabolism^28^,^29^. The application of enemas containing NAC has been shown to reduce inflammation in dextran sulphate- and acetic acid-induced colitis by removing ROS formed by the inflammatory process and increasing the activity of the antioxidant systems of the colonic mucosa^28^–^30^. However, despite the therapeutic efficacy of NAC in chemically induced colitis, its effects have rarely been tested in DC models^6^. Few studies reported that the intrarectal application of NAC reduced the mucosal inflammatory process, as well as the level of tissue oxidative stress, in an experimental model of DC^6^. Thus, clisteres with NAC could become a beneficial complementary therapeutic strategy for controlling the limiting symptoms of DC, improving the quality of life of people who already live with the difficulties of having a stoma. However, NAC has a low capacity of NAC to adhere to the colorectal mucosal surface excluded from transit required the substance to be applied by enemas several times a day, which reduced patient adherence to this therapeutic strategy^6^. It was from the low adhesiveness of NAC on the colonic mucosal surface that we had the idea of using it associated with the SCF molecule, which has a high capacity for mucosal adhesion.

This study revealed that the application of enemas containing isolated NAC reduced the inflammatory score, the level of neutrophil infiltration (as measured by MPO levels), and the level of tissue OS (as assessed by tissue MDA levels). The application of isolated NAC significantly increased the tissue levels of the antioxidant system components CAT, SOD, and GSH mainly in the first two weeks of intervention, which confirms the antioxidant power of the substance. The increase in antioxidant system components may have been smaller after four weeks because of the lower synthesis of these enzymes due to the deprivation of SCFAs, the main energy substrate for protein synthesis.

Only a single study previously evaluated the effects of a combination of enemas containing an emulsion of SCF and NAC in an experimental model of DC^14^. The authors showed that the combination of these two substances increased the production of mucins in the colonic epithelium, increasing the protection of mucosal cells against ROS^14^. Enemas containing a combination of the two substances was able to reduce the degree of mucosal inflammation, improving the healing of the excluded mucosa^14^. However, the authors did not evaluate whether the combination of the two substances had a better antioxidant effect than the effect of the two substances alone.

Considering that SCF and NAC have anti-inflammatory, antioxidant, and healing effects, the present study was designed to evaluate the ability of both substances to reduce inflammatory tissue damage and their antioxidant effects in the colonic mucosa lacking fecal stream. Compared with animals that received SCF or NAC alone, those that received an emulsion with both substances in combination presented significantly lower inflammatory scores, MPO and MDA than did those in the control group that received only saline. The serum levels of CAT and SOD were significantly greater than those in the control group but were similar to those in the intervention with the substances applied alone. In summary, the results of this study suggested that the use of enemas containing an emulsion of both substances can more effectively reduce inflammation and OS in colonic segments with DC.

However, this study has several limitations. First, this was a study in an experimental model of DC. Owing to ethical limitations, we used a small number of animals in each group. It is possible that with the use of a larger number of animals in each group, the results could be different. To minimize this problem, we used nonparametric tests to analyze the results. Since we conducted an experimental study on rats, the results should be interpreted with caution and only transposed to humans after conducting well-designed clinical studies. However, since both substances are already part of the therapeutic arsenal in humans and do not present serious adverse effects, studies in humans can be safely performed.

## Conclusion

SCF and NAC enemas alone or in combination can reduce inflammation, neutrophilic infiltration, and oxidative stress in colon segments without fecal stream.

## Data Availability

The data will be available upon request.
